# Common Genetic Variants Alter Metabolism and Influence Dietary Choline Requirements

**DOI:** 10.3390/nu9080837

**Published:** 2017-08-04

**Authors:** Ariel B. Ganz, Kevin C. Klatt, Marie A. Caudill

**Affiliations:** Division of Nutritional Sciences, Cornell University, Ithaca, NY 14853, USA; abg224@cornell.edu (A.B.G.); kck68@cornell.edu (K.C.K.)

**Keywords:** choline, genetics, nutrigenetics, dietary requirements

## Abstract

Nutrient needs, including those of the essential nutrient choline, are a population wide distribution. Adequate Intake (AI) recommendations for dietary choline (put forth by the National Academies of Medicine to aid individuals and groups in dietary assessment and planning) are grouped to account for the recognized unique needs associated with age, biological sex, and reproductive status (i.e., pregnancy or lactation). Established and emerging evidence supports the notion that common genetic variants are additional factors that substantially influence nutrient requirements. This review summarizes the genetic factors that influence choline requirements and metabolism in conditions of nutrient deprivation, as well as conditions of nutrient adequacy, across biological sexes and reproductive states. Overall, consistent and strong associative evidence demonstrates that common genetic variants in choline and folate pathway enzymes impact the metabolic handling of choline and the risk of nutrient inadequacy across varied dietary contexts. The studies characterized in this review also highlight the substantial promise of incorporating common genetic variants into choline intake recommendations to more precisely target the unique nutrient needs of these subgroups within the broader population. Additional studies are warranted to facilitate the translation of this evidence to nutrigenetics-based dietary approaches.

## 1. Choline Recommendations

**Figure 1:**
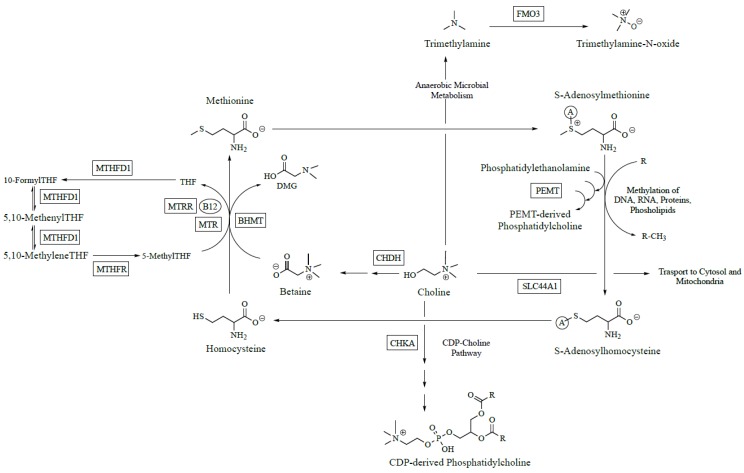
Simplified diagram of polymorphic folate and choline metabolic enzymes. Abbreviations: B12, vitamin B12; BHMT, betaine homocysteine *S*-methyltransferase; CDP, cytidine diphosphate; CHDH, choline dehydrogenase; CHKA, choline kinase alpha; FMO3, flavin monooxygenase isoform 3; MTHFD1, methylenetetrahydrofolate dehydrogenase 1; MTHFR, methylenetetrahydrofolate reductase; MTR, methionine synthase; MTRR, methionine synthase reductase; PC, phosphatidylcholine; PEMT, phosphatidylethanolamine *N*-methyltransferase; SLC44A1, solute carrier family 44 member 1; THF, tetrahydrofolate.

Choline is an essential nutrient that is converted to several metabolites with critical structural and regulatory roles within the body [1]. Choline can be (i) oxidized to betaine, an osmolyte and methyl donor; (ii) acetylated to form acetylcholine, a neurotransmitter involved in learning, memory, and attention; or (iii) phosphorylated and metabolized to phosphatidylcholine (PC), a critical component of cellular membranes that ensures fluidity and integrity (Figure 1). While humans can make choline endogenously via the hepatic phosphatidylethanolamine *N*-methyltransferase (*PEMT*) pathway (Figure 1), this route is not quantitatively sufficient to support biological requirements [2,3]. Thus, choline must be consumed exogenously to prevent symptoms of deficiency. In 1998, the Institute of Medicine (now known as the National Academies of Medicine) established an adequate intake (AI) for dietary choline [4]. For healthy adults, the AI was based on the amount of dietary choline needed to prevent a rise in liver enzymes associated with fat infiltration and liver damage. An AI is established when evidence is insufficient to determine the estimated average requirement (EAR), and therefore, cannot be used to estimate the probability of nutrient inadequacy for groups or individuals. This level of uncertainty about choline requirements has spurred substantial investigation of dietary choline demands throughout the life span. The results of controlled feeding interventions, summarized below, have yielded a complex picture of nutrient-genotype interactions which modify the response of choline-related metabolites and biomarkers to a wide-range of choline intakes.

## 2. Individual Differences in Choline Metabolism during Conditions of Choline Inadequacy 

The seminal study by Zeisel and colleagues which demonstrated that dietary choline is an essential nutrient for humans was published in 1991 and consisted of a highly controlled metabolic ward trial conducted in a cohort of healthy, young men [2]. All subjects were fed a semi-synthetic run-in diet containing 500 mg of choline per day for one week, and then randomized to continue the run-in diet or to a semisynthetic diet completely devoid of dietary choline for three weeks. Men consuming the choline deficient diet exhibited increased serum alanine aminotransferase activity, suggestive of liver dysfunction. Zeisel and colleagues further extended this work to include pre-menopausal and post-menopausal women to assess the effects of sex and life-stage influences on the susceptibility to frank choline deficiency [5]. In this follow-up trial, healthy men (*n* = 26 completed) and women (*n* = 31 completed) were confined to metabolic wards and given identical diets (isocaloric throughout the study). After ten days of calibration at 550 mg choline/day, the subjects were restricted to <50 mg of choline per day until they developed signs of organ dysfunction, indicating choline deficiency, or for a maximum of 42 days. While consuming the choline deficient diets, subjects were randomized to treatment with a folic acid supplement vs. consumption of only dietary folates. Subjects were considered to have choline deficiency-associated organ dysfunction if they displayed a greater than: five-fold increase of serum creatine phosphokinase (CPK) activity; a 1.5-fold increase in serum activity of aspartate transaminase, alanine transaminase, γ-glutamyl transpeptidase, or lactic dehydrogenase; and/or a 28% increase in liver fat content (assessed by magnetic resonance imaging) that was resolved after repletion of dietary choline [9]. When stratified by sex and menopausal status, 70% of men and 80% of postmenopausal women developed symptoms of choline deficiency, as compared to only 44% of premenopausal women [18]. While these results clearly implicated a role for female sex hormones in the susceptibility to choline deficiency, substantial variability still remained in the response to these highly controlled choline deficient diets. For example, within the male subset of the study, six participants developed organ dysfunction while consuming the baseline AI diet (550 mg choline/day), 14 developed signs of deficiency during the choline deficiency phase, and six did not develop signs of organ dysfunction at any time point.

Zeisel and colleagues hypothesized that common genetic variants impairing choline and folate metabolism would account for this variation in susceptibility to choline deficiency. Folate and choline metabolism are tightly linked due to their common participation in one carbon metabolism and ability to donate methyl groups to generate *S*-adenosylmethionine (SAM), the universal methyl donor. To test this hypothesis, the odds of developing choline deficiency were calculated based on the presence or absence of common single nucleotide polymorphisms (SNPs) in the genes for nine choline metabolizing enzymes [6,7] (Figure 1). Because choline and folate have overlapping metabolic functions, common SNPs in the genes for four folate metabolizing enzymes were also examined. For variants in folate metabolism, a 1958G→A (rs2236225) substitution in methylenetetrahydrofolate dehydrogenase 1 (*MTHFD1*) was associated with a seven-fold increased odds of developing organ dysfunction in individuals randomized to no supplemental folic acid; in premenopausal women who were carriers of this variant, the odds of developing organ dysfunction reached a striking 15-fold increase. No significant effects were noted for carriers of common variants in methylenetetrahydrofolate reductase (*MTHFR*) or the reduced folate carrier (RFC). For variants in choline metabolism, a polymorphism in the promoter region of *PEMT* (−744 G→C; rs12325817) increased the odds of developing organ dysfunction 25-fold, whereas an A→C substitution (rs9001) in choline dehydrogenase (*CHDH*), the enzyme which converts choline to betaine, yielded a protective effect. The lack of significant effects among other tested genotypes (e.g., *MTHFR*, *RFC*) on choline-deficiency associated organ dysfunction may be due to a true lack of functional significance or to the limited statistical power. For example, the sample in this study only included four homozygous individuals for each *MTHFR* SNP. The functional significance of variants within these genes requires further study.

The results of these analyses have spurred further investigative work to enhance current understanding of factors that influence choline needs. An estrogen response element (ERE) in the promoter region of the *PEMT* gene was confirmed, providing biological plausibility to the resistance to choline deficiency observed in premenopausal women [8]. Furthermore, common variants in the *PEMT* gene have been described which disrupt the binding of the estrogen receptor to this ERE and increase the dependence upon dietary choline to meet PC synthesis needs [9]. In a controlled trial where postmenopausal women were randomized to estrogen or placebo prior to consuming choline deficient diets, 73% of women receiving the placebo developed organ dysfunction, whereas only 18% of women receiving estrogen were susceptible [10].The high prevalence of common *PEMT* variants in the population [11] and their dose-dependent effects [11] on susceptibility to choline deficiency indicate the potential impact of taking genetic variation into account when assessing nutrient requirements. Table 1 provides a summary of SNPs associated with symptoms of choline deficiency under conditions of dietary choline deprivation.

## 3. Individual Differences in Choline Metabolism during Conditions of Folate Inadequacy

Work in the Zeisel lab has been formative in establishing nutrient-nutrient (i.e., between choline and folate) and nutrient-genotype interactions that influence the risk and onset of organ dysfunction in individuals deprived of dietary choline. The Zeisel lab’s work, coupled with earlier evidence from animal nutrition, suggested a strong interdependence between dietary choline and dietary folate needs; however, the impact of folate status and genetic variants in folate metabolism on choline needs outside of the context of frank choline deficiency required further exploration. To address this question, the Caudill laboratory undertook a controlled feeding study to assess the adequacy of the folate RDA in Mexican American men with the *MTHFR* 677C→T (rs1801133) 677CC or TT genotypes while consuming varied choline intakes [12]. The T allele exists at a high frequency in Mexican populations, and carriers were known to require higher folate intakes, but its relationship to choline was largely unknown at the time [13]. Men received identical diets containing 438 μg DFE/day and a total of 300, 550, 1100, or 2200 mg of choline/day; additionally, individuals in the 550 and 1100 mg/day groups consumed 15% of their choline intakes as choline chloride-(trimethyl-d_9_) for the last three weeks. While consuming the study diets, all participants displayed normal liver enzyme values, suggesting that 300 mg choline/day was sufficient in this population to prevent organ dysfunction. However, all treatment groups exhibited decreases in serum folate and an increase in plasma homocysteine, suggesting that the folate RDA is insufficient for men of Mexican descent; this effect was most pronounced in homozygous carriers of the *MTHFR* 677T allele, who exhibited a 170% increase in plasma homocysteine relative to an 18% increase in participants homozygous for the C allele (Solis et al, 2008). The plasma homocysteine response in the 677TT men exhibited high variation and further modeling of this response variable suggested that serum folate, plasma riboflavin, choline intake and the *PEMT* 5465G→A SNP (rs7946) collectively accounted for >80% of the variation [14]. Additionally, higher choline intakes (1100 and 2200 mg/day), but not lower intakes (300 and 550 mg/day), attenuated the rise in plasma homocysteine following a methionine load [15], suggesting that choline’s role in one carbon metabolism during folate restriction is most relevant during the postprandial state. Given choline’s use as a methyl donor, Caudill and colleagues further assessed the impact of choline intakes on whole genome DNA methylation [16]. Significant effects of choline intake levels were seen only in *MTHFR* 677CC carriers; the decrease in DNA methylation associated with compromised folate status was greatest in the 300mg choline intake group relative to the 1100 and 2200 mg groups.

The use of an isotope tracer is an important aspect of this study, as it allowed the Caudill lab to assess choline’s partitioning and flux through metabolic pathways. While observing static measures, such as metabolite pool size, can be informative, many metabolites are subject to homeostatic maintenance which can mask the impact of nutrient-nutrient and nutrient-genotype interactions on choline dynamics. Indeed, null associations between genotypes and static measures may lead investigators to question the functional importance of a specific polymorphism, while the impact of the variant may occur at the level of partitioning, recycling or metabolic flux. In the feeding study noted above, Mexican men consuming 550 and 1100 mg choline/day achieved similar unlabeled plasma betaine and PC concentrations; however, isotope data revealed a greater betaine-d_9_/PC-d_9_ enrichment ratio among men with the *MTHFR* 677TT genotype consuming 1100 mg choline/day relative to the 550 mg choline/day group (*p* = 0.03) [17]. Furthermore, men with *MTHFR* 677TT genotype displayed a greater urinary betaine to choline enrichment ratio (*p* = 0.04) and higher urinary d3-sarcosine enrichment. Collectively, these results suggest a greater turnover of choline to betaine and use of betaine as a methyl donor among 677TT men, as compared to 677CC men. Notably, the enrichment ratios for d3-PC and d-9 PC were not significantly different, indicating that greater use of betaine as a methyl donor did not affect the balance of PC derived from either the CDP-choline or *PEMT* pathways, a question of potential functional significance given their distinct fatty acid profiles.

Additional controlled feeding studies have been undertaken by the Caudill lab to assess the impact of nutrient-genotype interactions during folate inadequacy with fixed choline intakes. In a study by Abratte et al. [18], Mexican-American women with varied *MTHFR* C677T genotypes (*n* = 43; 13CC, 12CT, and 17TT) consumed 135 μg/day of dietary folate equivalents for 7 weeks, and were subsequently randomized to 400 or 800 dietary folate equivalents for an additional 7 weeks; choline intakes through this period were ~350 mg/day. Folate restriction led to decreases in plasma PC and sphingomyelin, with strong trends for this reduction in women with the 677CC or CT genotype, suggesting that the 677TT genotype plays a compensatory role during conditions of low folate intakes by shunting choline towards the CDP-choline pathway to maintain PC pools. A follow-up analysis of this study examined the effects of additional variants (*PEMT* rs12325817, *PEMT* rs7946, and *MTHFD1* rs2236225) on plasma homocysteine and PC levels [19]. Women with the *PEMT* rs7946 variant AA genotype and the *MTHFD1* variant AA genotype exhibited higher concentrations of plasma homocysteine during folate restriction. Additionally, women with the *PEMT* rs12325817 variant CC genotype exhibited an attenuated decline in circulating plasma PC. These results run counter to the hypothesis that *PEMT* impairing SNPs would compromise PC pools and reduce *S*-adenosylmethionine consumption and subsequent homocysteine levels. However, loss-of-function SNPs in *PEMT* may lead to a compensatory upregulation of the CDP-choline pathway, as seen in mice lacking *PEMT* [20], thereby reducing the supply of choline for betaine-dependent remethylation of homocysteine to methionine. Future well-powered studies, which assess the impact of these genotypes and their interactions, are needed to clarify the dynamic relationships between choline and folate nutriture, as these results are of high relevance to populations without folic acid supplementation.

## 4. Individual Differences in Choline Metabolism during Conditions of Nutrient Adequacy

**Figure 2:**
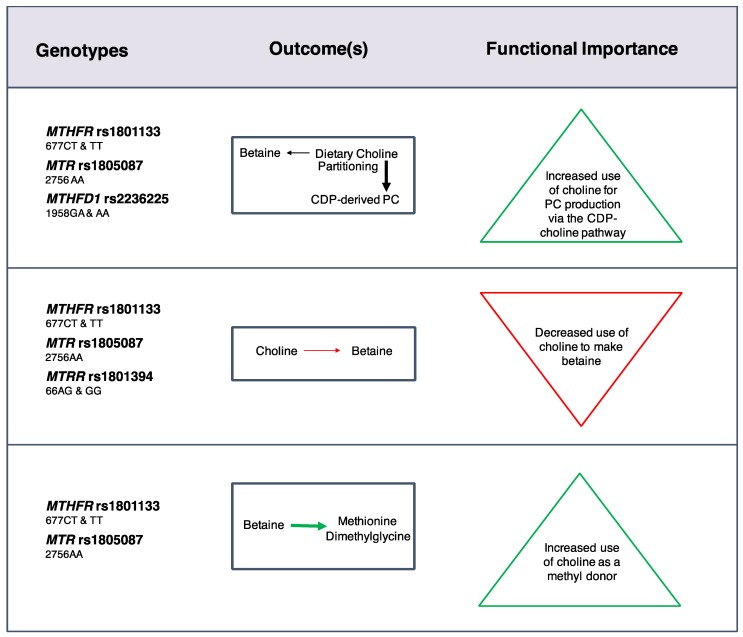
Effects of common genetic variants that impair folate metabolic enzymes on choline dynamics at adequate choline and folate intakes. Black arrows represent relative differences in the partitioning of dietary choline between metabolic endpoints. Green arrows and red arrows represent an increased and decreased flux of dietary choline, respectively, for a given metabolic outcome [23].

Early trials provided clear evidence that common genetic variants in folate and choline metabolism exhibit reproducible effects of a relevant magnitude during conditions of nutrient inadequacy. Most individuals in the U.S. population however, consume choline intakes well above those used in deficiency studies, and folate intakes are very high. Whether genetic variants are impactful during conditions of nutrient adequacy remains an active area of investigation. To address this research question, the Caudill lab has undertaken controlled feeding studies providing recommended levels of nutrient intakes in women across the reproductive life cycle [21]. Pregnant (*n* = 26), non-pregnant (*n* = 21), and lactating (*n* = 28) women consumed identical diets and a total of 480 or 930 mg choline/day for 10–12 weeks along with ~400 dietary folate equivalents, and 600 μg as supplemental folic acid (Yan et al, 2012). After week six, 22% of choline was provided as deuterium labeled choline chloride-(trimethyl-d_9_), a metabolic tracer that enables tracking of dietary choline throughout different metabolic pathways and compartments such as blood, urine, and breast milk. This allows for elucidation of the effects of various factors (e.g., reproductive state, choline intake, genetic variation) on the metabolic flux and partitioning of dietary choline [22,23,24]. With regard to genetic variation, Ganz et al. [23,24] assessed the impact of loss-of-function SNPs in folate and choline metabolizing genes. The folate SNPs, which included *MTHFR* rs1801133, methionine synthase (*MTR*) rs1805087 (wildtype), methionine synthase reductase (*MTRR*) rs1801394, and *MTHFD1* rs2236225, were all found to influence choline dynamics, frequently through interactions with reproductive state and choline intake [23] (Figure 2). Women with these variants partitioned more dietary choline toward PC biosynthesis via the CDP-choline pathway at the expense of betaine synthesis even when use of betaine as a methyl donor was increased. Choline intakes of 930 mg/day restored partitioning of dietary choline between betaine and CDP-derived PC among nonpregnant (*MTHFR* rs1801133 and *MTR* rs1805087 wildtype) and lactating (*MTHFD1* rs2236225) women with risk genotypes. Overall, these findings indicated that loss-of-function variants in folate metabolizing enzymes strain cellular PC production, possibly via impaired folate-dependent *PEMT*-derived PC synthesis, and suggested that women with these risk genotypes may benefit from choline intakes exceeding current recommendations.

**Figure 3:**
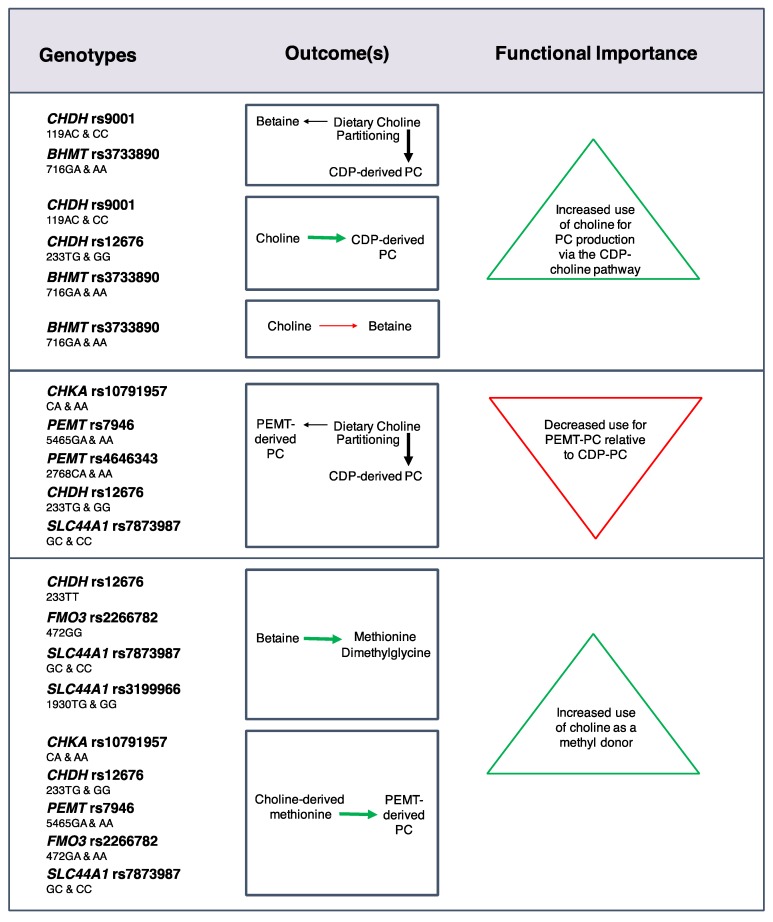
Effects of common genetic variants in choline metabolic enzymes on choline dynamics at adequate choline and folate intakes. Black arrows represent relative differences in the partitioning of dietary choline between metabolic endpoints. Green arrows and red arrows represent an increased and decreased flux of dietary choline, respectively, for a given metabolic outcome [24].

Effects on choline dynamics were also detected for SNPs in choline enzymes [24] (Figure 3). For example, the *CHKA* rs10791957 risk A allele was associated with a decreased use of dietary choline for *PEMT*-derived PC synthesis relative to CDP-derived PC synthesis, and decreased use of choline-derived methionine for *PEMT*-derived PC synthesis. Similar phenotypes were found for carriers of the *PEMT* rs4646343 T allele, the *PEMT* rs7946 T allele, and the *CHDH* rs12676 C allele. Differences in the use of choline as a methyl donor were also identified with greater use of choline-derived betaine as a methyl donor among women with the *FMO3* rs2266782 G allele, and non-pregnant women with the *CHDH* rs12676 A allele. Furthermore, women with the *SLC44A1* rs7873987 C allele and rs3199966 G allele exhibited a greater use of betaine for methionine synthesis in the higher choline intake group as compared to the lower intake group, suggesting that these genotypes readily utilize additional dietary choline for methyl donation. The partitioning of dietary choline between betaine and CDP-derived PC was also influenced by *CHDH* rs9001 and *BHMT* rs3733890. Overall, many of these SNPs appear to be associated with a differential need for dietary choline at adequate intakes.

Collectively, the results of this work establish an effect of common genetic variants on choline metabolism among individuals consuming nutrient-adequate diets. Numerous hypotheses can be generated from this data based on the identification of alterations in metabolic partitioning and flux. Further work identifying functional outcomes associated with these nutrient-genotype metabolic interactions will be paramount to translating these metabolic differences to genotype-specific dietary choline recommendations. Common outcomes such as decreased partitioning of choline towards the oxidative pathway (e.g., betaine) and alterations in PEMT-derived PC may well have functional consequences. For example, PEMT plays a critical role in exporting lipids from the liver to the bloodstream, and generates a PC molecule that is enriched in long chain omega 3 fatty acids such as docosahexaenoic acid [25]. Alterations in choline partitioning towards the PEMT pathway may have relevance for non-alcoholic fatty liver disease, hepatic and peripheral insulin sensitivity, atherosclerosis, and tissue docosahexaenoic acid availability [25,26].

## 5. Discussion and Implications

Complex, lifecycle-dependent, nutrient-nutrient, nutrient-reproductive state, and nutrient-genotype interactions clearly impact dietary choline needs, modify the risk of choline deficiency and alter nutrient metabolism. Notable common variants, such as those found in the *PEMT* and *MTHFD1* genes, appear to substantially modify choline needs during conditions of deficiency; furthermore, these and numerous other common variants throughout choline and folate metabolism alter the metabolic flux and partitioning of dietary choline during conditions of choline adequacy across reproductive stages.

A major goal of identifying genetic factors that influence choline needs is to inform dietary recommendations. Carriers of common genetic variants represent subgroups within the population whose risk of inadequacy is altered relative to the population mean; the studies characterized in this review highlight the substantial promise that the incorporation of genetic variants into dietary planning and recommendations may improve the assessment of nutrient inadequacy. However, at present, the translation of the results from these trials to nutrigenetics-based dietary approaches remains limited and substantial challenges remain for the field.

There is a need for well-powered trials that recruit individuals within genotypes, randomize to varied choline intakes, and assess causal impacts of these variants on functional outcomes. This need extends beyond just choline to the entire field of nutrigenetics. Randomized controlled trials have thus far been invaluable in the assessment of dietary choline needs and will be essential for the field of nutrigenetics and precision nutrition to advance; however, additional lines of evidence from observational analyses will also be key in assessing dose-response relationships, nutrient-nutrient interactions, the impact of co-morbidities, and long-term health outcomes. Currently, epidemiological analyses linking choline and other one-carbon nutrients to health outcomes are limited, and commonly do not consider the impact of genotypes, thus averaging across groups that may have distinct effects, and limiting the detection of functional health consequences associated with dietary choline intake. Given the impracticality of long-term dietary studies and concerns regarding exposure assessment and confounding in observational studies, future epidemiological analyses which utilize genetic variants as pseudo-randomized instrumental variables (i.e., Mendelian Randomization studies) may provide useful data for making causal inferences about the impacts of genetic alterations in nutrient metabolism. Several of these analyses exist already for the *MTHFR* C677T genotypes and numerous health outcomes [27,28]. If genetic data is to be incorporated into the probabilistic assessment of nutrient adequacy, strong causal evidence with meaningful functional outcomes will be necessary to support the cost and efforts associated with obtaining genetic information. Because complex interactions underlie dietary needs, including those between countless genetic factors, comprehensive network-based approaches to human trials will likely prove useful to aid in the incorporation of genetic factors into dietary requirements.

The current nutrient recommendations largely focus on outcomes related to the prevention of nutrient deficiency. Indeed, the choline AI was established to prevent elevated liver enzymes. However, numerous lines of evidence suggest that choline intakes may impact other outcomes as well. For example, dietary choline intakes higher than the current AI consumed during pregnancy may improve infant cognition [29,30] and positively influence pregnancy-related biomarkers [31,32]. However, the distribution of nutrient intakes in pregnant women required to achieve this outcome, and the functional impact of common genetic variants on pregnancy-related outcomes as well as fetal and infant health, remains largely unknown. It is highly likely that for some women, such as those with *PEMT* SNPs that render *PEMT* insensitive to the high estrogen milieu of pregnancy, choline needs are dramatically higher during the latter half of pregnancy. Furthermore, choline intakes have been implicated in the risk for several chronic diseases, including non-alcoholic fatty liver disease (protective) and cardiovascular disease (protective or detrimental) [4,26]. As the National Academies of Medicine shifts their strategy regarding nutrient recommendations to attempt to incorporate chronic disease outcomes into their framework, identifying dietary choline intakes that maximize health benefits and minimize risks will likely require knowledge of functional genetic variants. The task of stratifying dietary recommendations by genetic factors may seem daunting given the requisite increase in complexity of our conceptual understanding of nutrient needs. However, evidence supporting the sizable impact that genetic factors exert on nutrient metabolism and risk of deficiency provides optimism that nutrigenetics will ultimately limit controversial conflicting data, refining our ability to assess and manage individual nutritional needs.

## 6. Conclusions

In summary, there is consistent and strong associative evidence demonstrating that common genetic variants throughout choline and folate metabolism impact the metabolic handling of choline and the risk of nutrient inadequacy across varied dietary contexts. Additional evidence from large, well-powered, randomized controlled trials are needed to further define an estimated average requirement (EAR) for choline intakes across the lifespan, and clarify the utility of genetic variants in the assessment of nutrient inadequacy for both groups and individuals. Future dietary assessment and planning approaches that incorporate common genetic variants into this risk assessment hold substantial promise.

## Figures and Tables

**Table 1:** Summary of the associations between single nucleotide polymorphisms (SNPs) and the odds of developing choline deficiency- associated organ dysfunction (i.e., liver and/or skeletal muscle).

Gene	Function	SNP	Choline Deficiency Risk
*MTHFR*	Converts 5,10-methyleneTHF to 5-methylTHF. Rate limiting step in use of folate as methyl donor	rs1801133	NS
		rs1801131	NS
*MTHFD1*	Trifunctional enzyme that catalyzes the formation of 10-formyl-, 5,10-methenyl, and 5,10-methylene-THFs. SNP resides in the enzymatic activity associated with 10-formyl-THF synthesis.	rs2236225	↑ odds organ dysfunction
*RFC*	Transports reduced folates with a high affinity at a physiological pH.	rs1051266	NS
*MTR*	Vitamin B12- and folate-dependent conversion of homocysteine to methionine	rs1805087	Not evaluated
*MTRR*	Regenerates MTR after oxidation	rs1801394	Not evaluated
*CHKA*	Phosphorylates choline, first step in CDP-choline pathway	rs10791957	↓ odds organ dysfunction
*CHDH*	First step in oxidation of choline to betaine	rs9001	↓ odds organ dysfunction
		rs12676	↑ odds organ dysfunction
*BHMT*	Converts homocysteine to methionine using betaine as a methyl donor	rs3733890	NS
*PEMT*	Uses S-adenosylmethionine to triply methylate phosphatidylethanolamine to form PC (endogenous choline synthesis)	rs12325817	↑ odds organ dysfunction
		rs4646343	↑ odds organ dysfunction
		rs2266782	NS
*SLC44A1*	Transports choline across the cellular and mitochondrial membranes	rs7873937	↑ odds muscle damage
		rs3199966	↑ odds muscle damage

Abbreviations: *BHMT*, betaine homocysteine *S*-methyltransferase; *CHDH*, choline dehydrogenase; *CHKA*, choline kinase alpha; *MTHFD1*, methylenetetrahydrofolate dehydrogenase 1; *MTHFR*, methylenetetrahydrofolate reductase; *MTR*, methionine synthase; *MTRR*, methionine synthase reductase; PC, phosphatidylcholine; *PEMT*, phosphatidylethanolamine *N*-methyltransferase; *SLC44A1*, solute carrier family 44 member 1; THF, tetrahydrofolate. ↑ and ↓ indicate increased and decreased odds of developing organ dysfunction, respectively. Adapted from Ganz et al [24]. NS indicates that significant differences were not observed.
